# Laughter as ideological symptom: dialogical analysis of older adults’ discourse

**DOI:** 10.3389/fpsyg.2025.1606683

**Published:** 2025-06-13

**Authors:** Andrés Haye, Manuel Torres-Sahli

**Affiliations:** ^1^Faculty of Social Sciences, Pontificia Universidad Católica de Chile, Santiago, Chile; ^2^Center for Corporate Governance and Society, ESE Business School, Universidad de Los Andes, Santiago, Chile

**Keywords:** aging discourse, laughter, dialogical theory, ideological positioning, embodied enthymeme, discourse analysis, older adults

## Abstract

**Introduction:**

Laughter is increasingly investigated in ageing discourse, yet its ideological functions remain unclear. This paper examines how laughter functions as an ideological symptom in the discourse of older adults regarding aging. Drawing from Voloshinov’s dialogical theory, we conceptualize laughter not merely as an emotional response, but as an ideological symptom revealing tensions between what is said and what remains assumed.

**Methods:**

Four focus-group interviews with Chilean participants (*n* = 20; age 60–86) were analysed with a three-phase qualitative methodology combining thematic, dialogical and comparative techniques.

**Results:**

Laughter reliably marked interactional hotspots where talk about dependency, mortality and ageist stereotypes became sensitive. It acted as an embodied enthymeme that exposed—and regulated—tension between personal narratives and dominant ageing imaginaries. Gender and class shaped these patterns: women’s humour negotiated autonomy within family relations, whereas men’s joking resisted the figure of the “useless old man”.

**Discussion:**

Framing laughter as an ideological symptom shows how affect and normativity are related in later-life discourse, extending critical-gerontology debates and providing a replicable toolset for multimodal data.

## 1 Introduction

The aging of populations worldwide represents one of the most significant demographic transformations of the 21st century (World Health Organization, 2020). This shift raises fundamental questions about how societies understand, represent, and relate to older adults as social subjects (Grenier and Phillipson, 2018). While research has extensively documented the challenges of aging societies (Settersten and Angel, 2011), less attention has been paid to how older adults themselves articulate their experiences and position themselves as discursive subjects (Nikander, 2009). This gap is particularly significant given the persistent tendency in many societies to infantilize older adults or to treat them as unproblematic speakers whose discourse requires little interpretative effort—as if their expressions were either simplistically direct or merely symptomatic of decline, rather than complex discursive acts embedded in ideological fields (Salari, 2005; Hockey and James, 2003).

Within this context, understanding how older adults navigate and negotiate their subjective and social positioning through discourse becomes crucial (Bytheway, 2005). Especially relevant is their use of discursive resources—including non-verbal and affective elements—to articulate experience and either challenge or accommodate societal expectations (Coupland, 2001). Laughter, as both a bodily expression and a social act, emerges as a particularly rich site for examining how older adults manage the complex dynamics of aging discourse (Glenn and Holt, 2013). Yet despite growing interest in the discursive aspects of aging (Coupland, 2009), the role of laughter as an embodied enthymeme—pointing to shared understandings about aging that remain implicit—has remained critically understudied.

The study of discourse and aging has evolved significantly over the past decade, building on foundational work on language and identity in later life (Coupland, 2009; Ylänne, 2012) and expanding through cultural gerontology’s attention to subjectivity, embodiment, and the situated construction of aging (Twigg and Martin, 2015). Recent scholarship has increasingly questioned dominant “successful aging” paradigms for their individualistic assumptions and neglect of structural inequalities (Katz and Calasanti, 2015; Pickard, 2023). Particular attention has been paid to how aging is discursively constructed within what Gilleard and Higgs (2020) term the “social imaginary of the fourth age”—a culturally pervasive, often feared vision of deep old age marked by frailty and dependency. Within this evolving theoretical landscape, embodied expressions like laughter have gained new significance. No longer viewed as mere emotional reactions, they are recognized as sophisticated discursive moves through which older adults negotiate their positioning relative to aging stereotypes (Nimrod and Berdychevsky, 2018). However, despite growing interest in the multimodal dimensions of aging discourse (Brown and Prieto, 2021), the specific function of laughter as an ideological marker—a site where affect and social positioning converge—remains critically understudied. This gap is particularly significant in understanding how older adults from diverse cultural contexts, such as Chile, navigate the complex terrain between personal experience and societal expectations of aging (Pavez et al., 2023).

This limitation is especially relevant given that aging involves experiences that are difficult to express directly and that often challenge dominant cultural narratives about later life (Westerhof and Tulle, 2007). Topics like physical decline, social marginalization, or mortality frequently surface indirectly—through non-verbal cues, narrative disruptions, and notably, laughter (Glenn and Holt, 2013). While laughter in discourse has been extensively studied as a multifunctional social phenomenon (Jefferson, 1979; Glenn and Holt, 2013), with recent research demonstrating how older adults specifically employ humor to resist ageist stereotypes through strategies such as ‘distancing’ and ‘equalizing’ (Nimrod and Berdychevsky, 2018).

Voloshinov’s (1976, 1986) dialogical theory views utterances as responses embedded in an extra-verbal context—a shared social field that enables mutual understanding. This framework sees bodily expressions like laughter as integral elements of meaning-making that mark the boundary between what is said and what remains unspoken. It reframes ideology—not as a set of explicit values, but as the tension between shared assumptions and what speakers open to contestation through discourse. Recent Foucauldian analyses of aging discourse (Powell, 2019) complement this view by illustrating how older adults are positioned as subjects within dominant discourses, particularly in health and social care contexts, where aging is often constructed in terms of decline and medicalized in ways that can reinforce ageist stereotypes. This view also aligns with contemporary work on affect and embodiment in discourse (Wetherell, 2012; Gilleard and Higgs, 2015), where meaning is inseparable from relational positioning and embodied expression, and where the aging body is understood not merely as an object but as an active agent in constructing identity and navigating social expectations (Higgs and Gilleard, 2025).

This theoretical lens becomes particularly valuable for understanding laughter in aging discourse. When older adults laugh about their aging experiences, it often signals more than humor or social bonding. Laughter can mark moments where personal experience confronts societal expectations, where difficult truths about aging surface indirectly, or where implicit understandings momentarily become visible—without being explicitly articulated. Building on Voloshinov’s (1976) concept of the enthymeme—an argument that depends on shared, unstated premises—we propose that laughter functions as an embodied enthymeme, pointing to tacit knowledge and ideological positioning. In doing so, laughter reveals the discursive tensions that shape how aging is constructed and negotiated in everyday talk (Wetherell, 2012; Gilbert, 1997).

This study analyzes how laughter functions as an ideological symptom in the discourse of older adults regarding aging, based on four group interviews with participants over 60. By attending closely to the placement and function of laughter in conversation, we show how embodied expression can serve as a marker of ideological work in discourse—particularly in navigating themes of mortality and dependence. The paper first elaborates the theoretical framework, then details the methodological approach, and finally presents an analysis of the interviews. We conclude by discussing how our findings inform the study of aging discourse and contribute to broader debates on the intersection of affect, ideology, and meaning making in communication.

## 2 Dialogical approach to laughter in discourse

According to Voloshinov’s (1986) theory of discourse, language use is never ideologically neutral. Instead, discourse always unfolds within a field of social tensions, where communicative acts are shaped by interests and situated within a dynamic, contested cultural environment. Each time someone speaks, they are not simply conveying information but responding to prior voices circulating in a broader dialogical field. The meaning of what is said cannot be reduced to representational accuracy; it depends instead on the stance adopted, the interlocutors addressed—present, imagined, or absent—and the discourses that are taken up, resisted, or reconfigured in the process. This implies that the ideological function of discourse is embedded in its relational structure: Speech acts involve positioning oneself in relation to others.

Building on Voloshinov’s (1976, 1986) insights, we can argue that each utterance takes shape in close relation to its socio-material surroundings. What is said draws its intelligibility not only from its explicit content but also from a shared background of assumptions, experiences, and values that are left unspoken yet play a crucial role in making communication meaningful. Understanding an utterance thus requires access to a realm of tacit knowledge or a horizon of shared life that speakers rely on. These implicit frames of reference—what is presumed and usually not verbalized—form the scaffolding against which each communicative move gains its force. Rather than being external to discourse, this broader horizon enters the utterance as an integral part of its significance.

This view reframes ideology—not as a set of explicit values, but as the tension between shared assumptions and what speakers open to contestation through discourse. Ideology emerges in the friction between what is affirmed as given and what is posed as open to debate. The taken-for-granted is not confined to local common sense but may also reflect broader, systemic understandings that remain unexamined. Thus, each discursive move is situated in a web of alternatives, reinforcing some values while challenging or ignoring others. These values—understood as the implicit foundations of evaluative discourse—typically operate beneath the surface of explicit speech, inhabiting the space of the assumed or the non-said.

This unarticulated dimension of discourse does not function as an external cause or context; it is folded into the very structure of communication itself. The surrounding circumstances—material, cultural, interpersonal—are not passive backdrops but active components that shape and infuse the utterance with meaning. What is said is inseparable from how and where it is said, and from the field of expectations and valuations it addresses.

We can understand this dynamic through the rhetorical concept of the enthymeme. Aristotle (1991) described it in *Rhetoric* (1356b, I-II-9) as a form of persuasive, but non-demonstrative argumentation used in deliberative, political, and judicial discourse. While logically incomplete, the enthymeme relies on the audience to supply missing premises—thereby creating persuasive force and establishing social bonding. The notion of the enthymeme had been introduced before by Isocrates, but it is in Aristotle’s formulation that its argumentative role is most fully developed. Voloshinov repurposes this classical idea in a social key, suggesting that the utterances of everyday life function much like enthymemes: they presuppose a shared frame of meaning that is rarely questioned. “Every utterance in the business of life is an objective social enthymeme” (Voloshinov, 1976, p. 101). A mundane comment, a gesture, or a phrase may carry significant ideological weight precisely because of what it assumes rather than what it declares. When the surrounding context is unfamiliar or the shared background is absent, such utterances lose much of their force or even become unintelligible. They are, in a sense, like coded messages—‘passwords’—that only resonate within a particular social or cultural field. Voloshinov (1976) considers the enthymeme not as an abstract logical form, but as a finely tuned discursive move that draws on the assumed background of social life.

### 2.1 Laughter as a discursive phenomenon

The study of laughter within discourse analysis has drawn increasing attention, not only as a behavioral or psychological response but as a meaningful act embedded in social interaction. Discourse-oriented approaches treat laughter not as a mere byproduct of emotion, but as a situated communicative act—interpreted in context and within conversational flow. Pioneering work in conversation analysis, notably by Jefferson (1979), has shown that laughter is often jointly produced and interactionally managed. Speakers may signal the relevance of laughter through verbal cues, tone, bodily gestures, or prosodic shifts, and recipients respond in ways that either align with or resist these invitations. In this sense, laughter is not simply a reaction to a “funny” stimulus but part of the pragmatic structure of conversation—it can establish rapport, diffuse tension, or subtly challenge what is being said.

Further research (e.g., Glenn and Holt, 2013) has emphasized the complexity of laughter’s functions. People laugh in moments of ease as well as in moments of discomfort or ambiguity. Thus, the analyst’s task is not to decode a fixed meaning of laughter, but to examine what the laughter is doing—what kinds of alignments it produces, what assumptions it reinforces or destabilizes, and how it shapes the ongoing negotiation of meaning between participants.

The interactional organization of laughter in conversation has led researchers to distinguish between distinct types of laughter based on their sequential and pragmatic characteristics. While categorizations vary, three broad types can be identified: (1) invited laughter, where a speaker cues the listener to laugh; (2) volunteered laughter, where a speaker laughs independently of audience cues; and (3) sequential laughter, where laughter emerges as a response to an ongoing interactional dynamic. These distinctions help foreground the dialogical nature of laughter—not as an individual expression, but as a jointly negotiated act embedded in communicative positioning.

Invited laughter is the most familiar category. A speaker cues the audience to laugh through tone, exaggeration, or humorous phrasing. Here, laughter is not spontaneous but interactionally prompted—it serves to confirm a shared evaluation of the utterance’s content or tone.

Volunteered laughter occurs when speakers laugh without explicit cues, often as a way of managing discomfort, downplaying a statement, or signaling reflexivity. This laughter may not be directed toward others but rather function as a self-positioning device—marking an utterance as tentative, ambivalent, or emotionally complex. It is particularly relevant in conversations about aging, where older adults may invoke laughter to introduce sensitive topics without claiming full seriousness or emotional exposure.

Sequential laughter refers to laughter that arises within an unfolding interactional exchange—often triggered by an unexpected response, a conversational shift, or the cumulative tension of a narrative. It arises from interactional momentum rather than intentional design. This type of laughter can momentarily reframe a situation, disrupt expectations, or signal a shared shift in interpretive frame.

These three types are not mutually exclusive; in practice, they often overlap, with moments of laughter containing elements of invitation, voluntariness, and interactional emergence. Their analytical utility lies not in establishing rigid boundaries but in sensitizing us to the diverse roles laughter can play in discursive positioning.

### 2.2 Dialogic laughter

Dialogical theory of discourse emphasizes the relationship between laughter and normative instability. Bakhtin’s (1968) notion of festive laughter in the carnivalesque tradition is well known. It depicts collective, embodied, and transgressive forms that temporarily suspend hierarchies. However, his broader work reveals laughter’s more complex discursive functions.

Beyond its festive dimension, laughter serves as a multifaceted discourse marker that contributes to the organization of utterances within a dialogical field (Bakhtin, 1981). It allows speakers to step beyond literal content and take a social stance. This allows us to understand laughter as a suprasegmental marker that shapes and reveals evaluative orientation without necessarily doing so explicitly.

This discursive versatility of laughter—at once situated, affective, and evaluative—makes it particularly apt for capturing the implicit tensions and alignments that animate social interaction. Because it often exceeds explicit content, laughter grants access to what is otherwise unsaid in discourse. From this perspective, laughter can be approached not only as a pragmatic or rhetorical device, but also as an ideological symptom—one that reveals, in condensed form, the assumptions, exclusions, and evaluative frames that structure the field of interlocution.

Given its dependence on shared but often unstated meanings, laughter provides a particularly revealing entry point into the ideological dimensions of discourse.

According to Glenn and Holt, “an utterance may derive its laughability from taken-for-granted shared knowledge, memory, or understandings between speakers that may be invoked but remain obscure to analysts (and indeed to other participants)” (Glenn and Holt, 2013, p. 10). It is precisely this implicitness that allows us to treat laughter not merely as an interactional resource, but as a kind of symptom: a trace of underlying assumptions that remain unspoken, yet are made momentarily visible through the act of laughing.

This conception of laughter as symptom connects directly to Voloshinov’s notion of the enthymeme—that functions as a “password known only to those who belong to the same social purview” (Voloshinov, 1976, p. 101). From a dialogical and rhetorical standpoint, laughter can be read as an index of what is being positioned as absurd, inappropriate, excessive, or otherwise marked. Crucially, this positioning is rarely elaborated through argumentation. Instead, it is enacted through the affective force of laughter, bypassing overt justification.

In this way, laughter often functions as a shortcut to evaluation—anchoring agreement or disapproval without explicitly naming the terms of that judgment. What is laughable, then, tends to reflect what is ideologically recognizable as unworthy, excessive, or self-evidently flawed within a given social context.

Billig (2005) suggests that the primary effect of laughter is to ridicule—that is, to render something or someone as the object of scorn or dismissal. This ridicule often operates beneath the level of conscious reflection, reinforcing prevailing norms by designating certain views or behaviors as ‘obviously’ laughable.

In doing so, laughter contributes to the maintenance of ideological boundaries: it affirms what is to be taken seriously and what is not, who is ‘with us’ and who is not. Importantly, this process can occur without the laughable ever being named. The alignment between participants—confirmed through second laughter, for instance—signals that the underlying assumption has been recognized and accepted. But for analysts, the lack of overt articulation is precisely what marks this moment as ideologically significant: not through elaborate argument, but through tacit, habitual affirmations embedded in interaction. Thus, laughter becomes a discursive symptom of ideological positioning. It reveals how interlocutors navigate shared values, reassert common sense, and selectively open or close space for disagreement. By tracing when, how, and at what speakers laugh—especially in moments of tension, ambiguity, or rupture—we can begin to map the contours of the ideological field in which they are situated.

The critical analysis of laughter becomes not an inquiry into subjective emotions, but an exploration of how social values are reproduced, negotiated, or contested through embodied, affectively charged interaction.

Drawing from Bakhtin (1981) and Voloshinov (1986), we emphasized the inherently ideological nature of utterances: each is a situated response to a contested social field, shaped as much by what is said as by what remains unspoken. Meaning emerges not through representation alone, but through position-taking within an interactional and historical landscape of voices, values, and expectations. This conception leads us to a view of ideology as an ever-present tension between shared assumptions and contestable propositions. Rather than residing solely in the semantic content of speech, ideology takes root in the structure of addressivity and in the background of mutual understanding that renders utterances intelligible. What matters is not so much what is asserted as what is presumed, since such presumptions shape the possibility of speaking, responding, or remaining silent.

Within this frame, laughter has been examined not as a spontaneous emotional reaction, but as a discursive and relational act. It participates in the regulation of meaning and alignment in interaction, often operating as a subtle mechanism for reinforcing or negotiating shared assumptions. Laughter makes visible—if only fleetingly—the affective and evaluative contours of the ideological field in which participants are embedded. As such, it can be approached as a symptom: a moment in which ideology becomes perceptible in and through the embodied choreography of everyday talk. This theoretical approach opens specific possibilities for empirical analysis. In the next section, we outline a methodological strategy for applying these concepts to the study of group interviews. We describe how laughter can be used as an analytic cue to identify moments of affective and ideological salience, and how these moments can be unpacked to trace the configurations of meaning and positioning that emerge in discourse.

## 3 Method

### 3.1 Participants

This study draws on four group interviews conducted in Santiago, Osorno, and Machalí. Two groups were composed exclusively of women, one exclusively of men, and one was mixed-gender. Participants ranged in age from 67 to 86 years. We analyzed both the emergence of central themes and how participants subjectively positioned themselves in relation to them. Additionally, we examined overarching metaphors and implicit motivational structures that lent coherence to participants’ narratives and positioning moves.

Participants were recruited through local organizations—including municipal centers and cultural or recreational associations—and were members of pre-existing community groups. All participants volunteered after receiving an invitation and providing informed consent, and interviews were held in their usual meeting spaces.

The four groups were composed to ensure sociocultural homogeneity within each group—participants were acquainted with one another and had ongoing shared activities—while allowing for some variation across groups in terms of socioeconomic background, geographic location (metropolitan, small-town), and gender composition. Table 1 summarizes the composition and basic characteristics of the four interview groups.

**Table 1:** Characterization of interview groups.

Interview	Group	Location	Duration	Social class
1	5 (all women)	Osorno	60 min.	Upper-middle
2	8 (4 W / 4 M)	Machalí	150 min.	Lower-middle
3	3 (all women)	Santiago	100 min.	Lower-middle
4	4 (all men)	Santiago	120 min.	Middle

### 3.2 Interview design

The study employed a group interview format that combined techniques from focus groups and dialogical interviewing (see, e.g., Barbour, 2007; Frank, 2005; Wells et al., 2020). Each session began with a general prompt about aging and unfolded around a set of predefined thematic dimensions: (1) aging-related experiences and perceived differences in aging; (2) the relationship between aging, health, well-being; (3) notions of temporality and assumptions about the past and future, including bodily and generational perspectives; and (4) constructions of alterity, including inner interlocutors, experiences of otherness, and social relationships. The focus group structure encouraged each participant to share their views on key thematic areas, while the dialogical approach prioritized open-ended reflection and narrative development over question saturation or sequential structure. Interviews were designed to provoke collective discussion and create space for emergent storytelling within the group.

### 3.3 Procedure

The interviews were conducted by the same researcher, a female member of the study team, who also contacted and coordinated all four groups. In one of the interviews, conducted with an all-male group, the second author of the article was present as an observer, remaining on the margin and not participating in the conversation. The interviewer’s role was primarily to facilitate dialogue and, when necessary, to help connect emerging themes. A few hours after each interview, the interviewer wrote down her impressions and feelings during and after the interview, specifically about the context, special interaction moments, and own thoughts.

Interviews were audio-recorded, and the interviewer completed a reflective protocol during and after each encounter, noting subjective impressions of the interaction. These notes focused on affective dimensions of the dialogue, emotional climate, relational dynamics, and the role played by humor. Full transcripts were produced, preserving relevant features such as silences, interruptions, and laughter.

The study was reviewed and approved by the Ethics Committee of the School of Psychology at Pontificia Universidad Católica de Chile. The consent process invited participants to take part in a group interview with their existing older adult group, emphasized the voluntary nature of participation, and highlighted the value of learning about aging from the perspectives of older adults themselves. Participants provided written informed consent prior to participation.

There were no refusals to participate; on the contrary, all groups showed enthusiasm and a clear interest in sharing their experiences. A few months after the initial sessions, each group received a written summary of the content analysis of their interview and was invited to a follow-up conversation with the same interviewer. These meetings generated highly positive responses and contributed additional insight to the ongoing analysis. Finally, participants received a letter containing a report summarizing the study findings.

### 3.4 Data analyses

Our methodological approach was structured in three analytical cycles, each oriented toward a different dimension of the material. Throughout all phases, repeated engagement with the data was enriched by dialogue within a multidisciplinary research team, contributing to theoretical and interpretive depth.

The *first cycle* involved a thematic analysis aimed at mapping the discursive contents that emerged across interviews. This phase generated guiding questions organized around three central axes: the sociocultural framing of biographical experience, the psychological structuring of aging, and the articulation between subjectivity and health. The analysis employed content analysis techniques inspired by grounded theory (Strauss and Corbin, 1997), with the goal of establishing a semantic field to support deeper interpretive work.

However, thematic mapping alone was insufficient to grasp the interactional logic and discursive structuring at play. Recognizing interviews as discursive events—not mere repositories of pre-formed beliefs—we conducted a *second analytical cycle* focused on discourse as position-taking. Here we considered both formal and material aspects of enunciation (Haye and Larraín, 2011, 2013; Linell, 1998), attending to how subjective and normative positioning unfolds through discourse. While content analysis remained present, it was reframed as one among several clues for interpreting discursive formations as situated rhetorical projects (Larraín and Haye, 2012).

In this cycle, interviews were reanalyzed from a dialogical perspective, with specific attention to affective dynamics, the interviewer-interviewee relationship, constructions of alterity, normative tensions, and paralinguistic cues such as silences, repetitions, interruptions, tone, rhythm, and—centrally—laughter. Laughter emerged as a particularly fertile site for tracing the intersection between affect and ideology within discourse. As a pragmatic and affective marker, humorous laughter pointed to moments of evaluative salience, where assumptions were affirmed, questioned, or displaced. Thus, we conducted a systematic analysis of laughter episodes, examining their sequential placement and rhetorical effects. In each case, laughter was treated not as a peripheral feature but as a discursive operator that helped organize and condense positioning efforts. Through laughter, participants often marked shared assumptions, registered discomfort, or managed the boundaries of what could be said and what remained implicit.

The *third cycle* involved a comparative analysis across all interviews, focused on how comic laughter operated in relation to processes of affective alignment, the construction of otherness, and the negotiation of norms. We analyzed a selection of particularly revealing laughter events to understand how older adults navigated moments of discursive ambiguity, tension, or implicit conflict. In this way, laughter was approached as a discursive symptom—indexing not only shared meanings, but also the contours of what remained unsaid. This phase offered insight into how speakers grappled with the ideological assumptions that underpin their narratives on aging.

Our approach to discourse analysis builds upon recent methodological innovations in the study of aging discourse. Varela-Suárez (2024) provides a structured framework for analyzing discourse in older populations, emphasizing the importance of contextualized, pragmatic analysis that attends to both explicit content and implicit meaning-making. Following Phelan’s (2018) guidance on researching ageism through discourse, we attend to how taken-for-granted “truths” about older people are reproduced, contested, or negotiated in conversation. This includes analyzing not only what is said but also how it is said—with particular attention to paralinguistic features like laughter, which often mark moments of heightened ideological significance. Our three-phase analytical approach aligns with contemporary recommendations for studying the multimodal dimensions of communication (Brown and Prieto, 2021), recognizing that meaning emerges not only through verbal content but integrating vocal, gestural, and affective cues. This methodological orientation allows us to capture how laughter functions as what Voloshinov terms a “social enthymeme”—a communicative move that relies on shared but unspoken assumptions—particularly in the Chilean context where aging experiences may reflect both global and culturally specific ideological tensions (Pavez et al., 2023).

## 4 Results

A first analytical approach sought to identify commonalities across interviews, groups, and individuals in the discursive content that emerged when discussing aging. This analysis was previously reported in a study that explored the subjective dimension of aging—how subjectivity is articulated in older adults’ discourse and how resistance to the internalization of aging is constructed (Haye et al., 2022). The study described how older adults in Chile negotiate their personal and age-related identity within a dilemmatic sociocultural framing of aging, marked by ambivalence and subjective struggles. A particular repertoire of discursive elements emerged, shaping the way aging was verbalized in the interviews.

This article focuses on the second and third analytical cycles, which took a radically relational approach to the affective and ideological aspects of participant positioning throughout the interviews—viewing each interview as a unique collective utterance. The key results presented here derive from this dialogical perspective, focusing on the role of laughter as a multifaceted discursive marker of ideological positioning, through which older adults actively construct meaning from within the continuous differentiation of the explicit and the implicit. The specific question we address is how laughter helps understand the positioning moves that shape the collective contribution of each group.

For each interview, we present a synthesis of findings integrating different analytical levels to account for a unique narrative: (1) affective tone and interviewer reflexivity, (2) paralinguistic features (rhythm, silences, interruptions, laughter), (3) thematic content and positioning, and (4) normative discourse. Social and gender backgrounds were considered in the analysis of each interview and, then, in their comparison.

### 4.1 Interview 1

The overall tone of the interview is fluid yet somewhat superficial, with a fast-paced rhythm due to time constraints (approximately 1 h). On an affective level, the conversation does not delve deeply into emerging topics, primarily due to the limited time available. However, for the interviewer, the experience is both comfortable and rewarding, as it provides a rare opportunity to listen to older women from her own province—something often hindered by Chile’s strong centralization. Despite this sense of familiarity, a subtle yet affectionate hierarchy is present; the interviewees position the interviewer as a younger “other,” occasionally highlighting how unfamiliar younger generations can seem to them. This dynamic is balanced by the shared connection to Osorno, which fosters a sense of closeness. In sum, the affective tone of the interview is one of warmth and enthusiasm, shaped by the participants’ appreciation for being heard.

The analysis of paralinguistic aspects of discourse—particularly tone and rhythm, silences, and interruptions, and especially laughter, as indicators of interactional dynamics—reveals an irresistible urge to talk about the interview topic. In this group, there are no silences; participants often speak over one another, interrupting to engage rather than derailing the conversation, reinforcing a dominant voice that continues its course. Laughter, in turn, frequently arises when painful or embarrassing truths about aging are expressed, especially regarding (lack of) control and (loss of) autonomy. In this interview, humor is linked to the challenge of striking a balanced stance toward an externalized concept of aging. For example, after 20 min of the interview:

- *I do not consider myself old.*
- *(Interviewer) How would you describe an old person?*
- *An old person? An idiot.*
- *Wrinkled [laughter], you know, slow.*
- *A decrepit old person—that’s what being old means to me.*
- *(Interviewer) Right, and for you?*
- *Look, it took me a long time to even accept being called a grandma.*
- *(Interviewer) I see…*
- One time, my car broke down in the street, so I got out to check, and this young guy on a bike passed by and said, “Granny, your car broke down!” And I thought, damn… I’m *granny* now? [laughs] That’s when I felt old.

This example illustrates how important is, for the concrete discursive becoming of this interview, the perspective or discourse of the other, laughing first when using a negative stereotype to externalize aging, and then directly quoting the words of the other. In addition, the example shows that two discourses or positions are entangled, the “realistic” position (of the other) towards accepting aging effects, and the “positive” position about activity and vitality, a discourse that is taken from the other but with a sensible effort/difficulty to assimilate it, at some point expressing resistance. Interestingly, these constructions of otherness and position-taking efforts reveal particularly unstable and ambiguous during the interview.

Indeed, the structural content analysis performed in the first cycle revealed that one of the dominant topics in this interview is the profound shock of recognizing and understanding themselves as old. This realization is deeply shaped by external voices that produce this news, as from the outside, although it is in their own discourse the interviewees make this exteriority relevant and materialized in social others. Yet, at the very beginning of the interview: “*When you are driving and want to renew your license, but they will not give it to you because you are old [Laughs]*.” The comic and laughable here does not derive only from the crude expression, which represents and eventually exaggerates the discourse of the other; in addition, it is possible to think that the expression is comic and crude because it confronts the subjective position of the speaker and peer listeners, affect-as-desire, on the one hand, and affect-as-passion, that is, the discursive position of accepting dependence and death, represented in the voices of social alterity (family, state, or aging driving norms).

In this dialogical context, family becomes essential—positioned as the guarantor of a “good old age,” the source of both emotional and financial support. However, this guarantee is not unconditional; it depends on maintaining a good relationship with family members. The interviewees acknowledge the need to establish an “optimal distance” with those closest to them, primarily their children and grandchildren. This distance is rooted in both love and respect—for their relatives’ choices and way of life. They do not wish to be absent, but neither do they want to overstep with an all-encompassing presence. Striking this balance is their key strategy for ensuring a good old age. An active effort or obstacle to balance is needed, though, because a distance tends to take the form of a temporal gap. A recurring theme is a sense of disconnection from younger generations, often tied to feeling left behind: “They’re all studying, they are all working,” a phrase that subtly conveys exclusion.

Similarly, functionality and activity are highly valued: *“What do you think enables healthy aging?” / “Activity.” / “Activity.” / “Activity—reading…”* The interview also reflects an ambivalence about activity: it is seen as essential, yet also as a burden, as aging is often perceived through the metaphor of a deteriorating machine, reinforcing an urgent need to remain “functional”: *“But it’s your battery that runs out, not the car’s.”* The drive to persist and remain engaged is not only about avoiding isolation—*“Do not shut yourself in”*—but also about how they are perceived by others. Approval or disapproval signals what is considered acceptable, making autonomy and independence almost an unquestioned expectation: *“I think as long as you are healthy and can manage on your own” and “My ideal was always to not be a burden on my children.”*

Normative expressions appear frequently in the interview in various forms. Some take the shape of self-imposed rules or common-sense notions of how one *should* be, which the interviewees apply to themselves: *“You should not shut yourself in, you should not isolate yourself,” “One must find ways to stay entertained.”* The analysis of normative aspects of discourse reveals that semantic shifts are rare in this interview, but when they do occur, they serve as attempts to stop or redirect difficult or painful narratives. In this sense, semantic shifts function as a form of self-censorship:

- *Hey, last year in our group, how many passed away? Three?*
- *Yes, yes…*
- *Normita.*
- *And Tuti too…*
- *Yes, Normita.*
- *Normita always came, but Tuti did not…*
- *Well, at that age, what can you expect…*
- *Hey, and Tuti…*
- *They were all around ninety.*
- *And…?*
- *I went to the Hogar de las Socorras… Oh, what a marvel!*
- *There are only two there who are still holding up at ninety.* [Laughter].

In sum, these key themes of the interview are configured around a central tension mobilizing the discourse about aging in this group: autonomy versus dependence. The interviewees associate autonomy with the ideal of a “good old age,” defining it as the ability to move freely and carry out daily routines without assistance. Autonomy is understood as both a physical and mental condition that enables them to stay active independently. Dependence, in contrast, is perceived as a sign of “bad aging” and a potential burden on their families. The primary form of otherness in this group’s discourse revolves around family and younger generations, both perceived positively as sources of support, care, and intergenerational interaction. However, there is also a sense of estrangement from the young; despite being part of their family network and providing support, younger generations are associated with a way of life that feels increasingly difficult to understand.

What is particularly significant in this dialogical field is how laughter functions as a symptom of this tension—emerging precisely when participants momentarily recognize and yield to the discourse of the other, marking these instances with particular affective intensity.

Humoristic turns in this group predominantly revolve around accepting a minimum to losing self-control and independence, highlighting that their discourse becomes as a particular organization of the others’ discourses, inner alterity with whom—along with and against which—they raise their own positioning. At minute 24:

- *I think deafness is because young people do not pronounce properly, they do not articulate well.*
- *That’s just an old people’s excuse, come on! [Laughs].*

Laughter comes when a sort of truth is revealed, against the older adult’s resistance to the “realistic” discourse of others about the effects of aging. Participants laugh here at the moment there is a gesture of acceptance. In contrast to the enthusiastic tone of the interview, the dialogical analysis finds an important effort of resistance, marked a resigned acceptance of reality—one that carries a sense of resistance but ultimately feels like a defeated complaint: *“I think sometimes you just have to accept things and not be too demanding, because if the kids cannot take care of you or aren’t around, well, they just aren’t. You grab a book, watch TV, you just have to accept it.”*

This exemplifies what Billig (*2005*) describes as the ideological function of humor—not merely expressing emotion but organizing social relations through what remains unsaid. Within this ambiguous space, aging gains meaning for these women through a complex negotiation of position-taking that is both with and against the discourses of others. Their subjectivity is shaped by this dialogical process, from which they construct a discourse on preparing for a “good old age”—one that resists the ultimate other, which is the loss of physical and mental functionality, anticipated as the loss of personal independence.

### 4.2 Interview 2

Of the four interviews conducted, this was by far the most engaging, holding the interviewer’s attention throughout, as it reached a greater level of depth than the others. This interview lasted more than 2 h. The emotional tone of the interview fluctuated: at times, nostalgia dominated the discussion; at others, frustration and anger surfaced as participants criticized what they saw as an unjust reality for older adults’ post-retirement. Moments of humor also played a key role, providing relief after emotionally charged discussions and serving to voice difficult truths. Overall, the group was cohesive and eager to share as much as possible.

The analysis of paralinguistic aspects in the discourse reveals a recurring denial of death in this group, expressed through abrupt thematic shifts, interruptions, and laughter—particularly in moments of lightheartedness, playfulness, and even childlike humor. However, at times, the discourse also takes on a dramatic and serious tone, with blunt self-reflections expressed without hesitation: *“It’s the past now, there’s no point in regret.”* This highlights an ambivalent attitude toward aging within the group.

For these men and women, aging is understood in two distinct ways, as a natural process and as an external imposition. For some, aging is simply another stage of life—something natural and continuous: *“I do not feel old, I’m just living another stage.”* However, they also recognize that aging is externally imposed, a category dictated by institutions: *“The law sets a date, and from that moment on, I have to transition into a different age category.”* The tension between these perspectives shapes their experience of growing older. *“Aging means I have to adjust, I have to change roles because society demands it. And besides, the law sets a date, and from that moment on, I have to transition into a different age category.”* According to them, the workforce (and productivity in general) is one of the areas most affected by this categorization, as it comes with restrictive practices and new living conditions. They feel stripped of what they have earned through work and effort, relegated to a state of total exclusion—a space of “extermination” and “nonexistence.”

However, the content analysis also highlights the need of “preparing” for the aging process: *“Preparation is essential—just like a non-athlete who wants to run a marathon, we all have a marathon ahead of us as older adults. So. older adults must prepare, they must be prepared to run that marathon, physically and psychologically.”* Likewise, aging for this group is deeply tied to the weight of accumulated experience. They see passing down their knowledge as both a right and a responsibility, yet it often brings frustration rather than fulfillment. *“There’s so much that needs to be passed on, it’s overwhelming,”* one participant notes. The urgency to share, paired with the feeling of being unheard, creates a sense of desperation: “Older adults are desperate to share more. That’s the truth.”

Consistently, the analysis of normative aspects—such as imperatives and authoritative voices—shows the presence of self-imposed mandates. These take the form of personal obligations aimed at maintaining an active lifestyle through self-management of personal resources: *“I believe it’s essential that we prepare ourselves—or that older adults be prepared—to run this marathon, both physically and psychologically.”* This suggests that these self-imposed rules revolve around strategies for staying active, ensuring the continuation of a lifestyle they seek to prolong.

Other normative expressions appear in discourse depending on the intended audience. When addressing younger generations, authoritative voices emerge in the form of advice, conveying what they consider a proper way of life. In contrast, when speaking about the government, these voices become imperative, demanding greater attention and care for the elderly.

### 4.3 Interview 3

The overall tone of the interview is that of a fluid conversation, filled with laughter and a sense of female camaraderie. From the interviewer’s perspective, there was a strong degree of alignment with the dominant discourse of liberal capitalism, which frames “positive aging” as a standard and provides means to maintain the body and health in an actively vital state. As a result, at times, she found it challenging to stay engaged and focused on the conversation. Additionally, there are moments of underlying frustration, subtly conveyed through dismissive remarks directed at the interviewer.

The thematic analysis of the interview reveals that the conversation is structured around four main topics related to aging:

- Aging as an unconscious process, one that individuals do not fully perceive as it happens: *“I do not know… I just have not even realized when I started aging.”*
- Aging as a period of exclusion, where they feel unable to participate in new spaces or use technologies that emerged during their later years: *“Unfortunately, things have changed for the worse. Things have changed for the worse. Technology? I could not care less about technology. I barely even use my cellphone.”*
- T*he “adjustments” demanded by aging, requiring individuals to navigate a stage where self-care and affection become increasingly important: “We have to monitor our health, go to check-ups, do what the doctor says, and watch our diet—because diet is very important, very important.”*
- The experience of taking on a new role within the family—one that requires maintaining a certain distance from relatives while still depending on them emotionally: *“Love the grandkids and all, but only up to a point.”*

For these women, aging is not something they consciously experience. *“I just have not realized when I started aging,”* one participant confesses, while another adds, *“If I did not look in the mirror and see my wrinkles, I would not even notice.”* They see old age as a continuation of previous life stages, a process that unfolds without clear demarcations. *Although they do not perceive themselves as old, often citing their physical well-being: “Of course! If I did not look in the mirror and see my wrinkles, I would not even notice. Other than that, I can do any exercise you ask me to do, and I’ll do it.”* They even bring about positive outcomes of aging, but not without exceptions that represent the ambivalence about aging:

- *Oh, I’m the complete opposite! I get so much attention! On the bus…*
- *Same here!*
- *I get on the subway, a man stands up, gives me his hand, sits me down [laughs]. You know, sometimes I even feel embarrassed because up to three people stand up to offer me their seat!*
- *I do not get such nice treatment. [Laughs].*

At the same time, they express discomfort with the present era in which they have grown old—an era they view negatively: *“Because, you know, there’s all kinds of things in life, and young people today are so different; they take different paths. And that affects families too, and in the end, the ones who suffer the most are older adults.” The interviewees perceive their role in society as increasingly marginal. When asked about the place of older adults in Chilean society, they respond with humor:*

- *Last place. [Laughs].*
- *Second to last, you say? [Laughs].*
- *No, it’s just that I put myself right in the middle—like a decorative vase! [Laughs].*

Laughter sometimes helps in accepting the effects of aging, particularly when framed through the lens of others’ discourse: *“Imagine, I’m deaf—seriously, I’m deaf! And on top of that, things like air conditioning bother me, it gets to me and makes it harder for me to hear you. But I’ve taken it all in stride, you know? I’m about to turn 85—what more can I ask of my ears? [Laughs].”*

- *Shuffling your feet—that’s old age.*
- *That’s old age.*
- *Oh, I shuffle my feet too! [Laughs].*

The analysis of paralinguistic aspects of discourse reveals that laughter occurs frequently throughout the interview. It emerges primarily in moments of playfulness, flirtation, and lightheartedness, serving as a way to turn bitter topics, such as aging, into humorous situations: *“Damn, I’m getting old, I say. But then I wash my face, fix myself up, and that’s it—I’m ready. [Laughter].”*

Laughter also emerges when discussing aspects of old age, particularly its disadvantages. When one participant makes a confession about aging, it often triggers laughter from the group:

- *Yeah, old folks are always sitting around…*
- *You cannot dance to one of these cumbias; you have to stay seated.*
- *Or just get up and move, that’s it! [Laughter].*

Interruptions, in turn, serve to reaffirm their positions as older adults. Rather than diverting the conversation, these interruptions reinforce certain viewpoints, seemingly as a defensive stance. They act as echoes of a central voice, strengthening the argument being developed:

- *Of course. But now, I tell them, I come here, I’m not in a rush, I left my lunch ready, whatever, and I can wait, just like you, I tell them, I can wait. So no, do not worry about me. ‘But you should ask…’*
- *No, I tell them, I’ll wait. Because you might need it more than I do.*
- *Of course.*
- *The point is that I [interruption]*
- *It’s called being aware, being a conscious person.*
- *Of course. Absolutely. I have to respect her.*

Similarly, the analysis of the normative aspects of discourse reveals that this group expresses self-imposed rules. These serve as internalized mandates, guiding what they must do to achieve certain goals and maintain a sense of well-being. Some of these self-impositions focus on adopting a particular mindset and attitude toward life: *“One must think that these are just things that happen in life,” “One must be positive.,” “One must always be happy.”*

Others emphasize the need to accept past or future events in order to face them, even if only with resignation:

- *One must accept the passing of the years, enjoy life, not dwell on things, and get along with everyone.*
- *Accept that what’s coming in the future may not be very good.*

Another set of self-impositions is aimed at maintaining an “optimal distance” from family, ensuring they occupy what they perceive as an appropriate role in their new stage of life: *“That’s what one has to be. One must be useful, not intrusive. One must not interfere.”* Additionally, they impose upon themselves the duty to follow externally prescribed care routines: *“So we must take care of our health, go to check-ups, and do what the doctor says.”* Lastly, some self-imposed rules focus on self-regulation, adapting their behavior to the physical limitations of aging: *“You have to start slowing down. Little by little. I used to get all the way over there; now I’ll just go up to here.”*

*In this context,* the interviewees highlight a series of adjustments they make to cope with aging. One of these is shifting the care they once provided to their families toward caring for themselves: *“You can get sick, but like I said before, you have to love yourself and take care of yourself, because we have already given everything we had to give.”* Another key adjustment is maintaining good relationships with family members. This involves being autonomous while still depending on them emotionally: *“Because as the years go by, what you need the most is love. More than anything. Not gifts, not things… Just love.”* At the same time, emotional dependence on family is shaped by a sense of reciprocity—the belief that past kindness and care will be repaid: *“Look, I think that sometimes—not always—you get back what you give. If you tried to be a good person when you were young, if you treated your parents and family well, sometimes that comes back to you.”*

Laughter in this group emerges not only when participants implicitly accept aspects of dominant discourses on aging, but also when the violence of such discourses is being resisted or exposed:

- *We’ve been bouncing around with this really ugly idea. There was this show once on Channel 13, a family of dinosaurs—this chubby little baby dinosaur—sometime around 1994 or 1995. And in the last episode, the whole family was getting ready to say goodbye to the grandma. The grandma was like, ‘Well, it’s my time, I’m going now,’ and all that. Long story short, the family heads out, walks through a valley, and comes to this cliff—just like this one—‘Bye, grandma…’.*
- *[Interviewer] Did they throw her off?*
- *Yes. (Laughter).*

### 4.4 Interview 4

For the interviewer, this interview was engaging and entertaining, with humor playing a central role and laughter being a constant presence. However, she noted feeling uncomfortable toward the end due to openly sexist remarks directed at women, particularly regarding their appearance and self-care. This discomfort led her to respond at times with surprise or implicit disapproval, using humor to challenge these comments. It seems that, at times, the sharp criticism of women functioned as an indirect way for the participants to talk about themselves. Within a machista framework, women are framed as sensitive and vulnerable, whereas masculinity is denied the same space for openness. As a result, the emotional tone of the interview remained superficial, even trivial. Moments of deep reflection were rare, and the conversation was dominated by anecdotes.

The analysis of paralinguistic aspects of discourse reveals that within this group, a cheerful and lighthearted tone often masked a difficulty in discussing certain topics. These include the experience of aging as the daily reality of social displacement as “old people” and the looming presence of death—acknowledged in relation to others but simultaneously denied when it comes to oneself, and also as a physical process: *“The years do not pass, they do not pass… they just pile up [laughter].”*

A central theme in this interview is aging as an accumulation of experiences that gradually become a burden: *“That’s the truth. It feels like you are filling up a backpack, and then you feel the weight on your shoulders.” The analysis of paralinguistic aspects of discourse reveals that within this group, a cheerful and lighthearted tone often masked a difficulty in discussing certain topics. These include aging as a daily experience of social displacement as “old people,” and the looming presence of death—acknowledged when speaking of others, yet denied when speaking of oneself, both socially and physically: “The years do not pass, they do not pass… they just pile up [laughter].”*

Other key themes include the need to share accumulated experience as a way of “giving back” or “passing down” knowledge, and the importance of staying active—not just to feel useful, but to keep death at bay: *“That’s the ‘useless old man,’ in quotes. But someone who stays active, a man who keeps busy, who comes home and says, ‘Look, I painted this, I hammered in this nail, I hung this picture’—you know he’s alive.”*

The analysis of normative aspects of discourse reveals that these themes are addressed or responded to with self-imposed imperatives, primarily emphasizing the need to stay active and maintain social connections to push back against aging—and death. Aging is frequently framed as a process that must be resisted. Participants stress the importance of staying active and socially engaged, warning against the dangers of withdrawal: *“That’s it. They shut down, do not want to do anything. That needs to be avoided… They just close themselves off, will not accept anything, until they die.”*

These normative voices shift outward, typically toward the State or institutions, insisting on the importance of “passing on” experience—particularly to younger generations. They call for institutional spaces that would allow this exchange: *“We should recognize that all the time we have lived, the people we have shared with, all the experiences we have accumulated—when we reach old age, we should be able to reflect on them and pass them on to those coming after us.”* Passing down or “transmitting” this accumulated experience becomes essential, as leaving a legacy to future generations—almost by accident, since their peers are also seen as worthy recipients—offers a form of relief. The act of sharing experience is thus associated with a sense of fulfillment, perceived as mutually beneficial: *“Being able to support other older adults, as an older adult myself—being able to give, for example, just by lending an ear to someone who is alone, by listening, looking them in the eye… And then you realize: I give them my time, but they are also giving me something I lack,” “But I believe that we, for instance, can contribute experience—because, as they say, experience is the mother of science. This is a stage of life where we can do things, not just for ourselves but also for the benefit of others.”*

In this connection, younger generations emerge as a specific “other.” They are the intended recipients of this legacy, yet they also deny older adults their role as valid interlocutors, pushing them aside as voices of authority. This relationship, then, is rendered impossible. On one hand, older adults envy the vitality of youth; on the other, they perceive them as lacking depth, believing that only the accumulation of years ensures true maturity: “I have great envy for young people, but no, I’m not angry with them. *Usually, older people are angry at the young—because they are young, because they move, because they have different attitudes. But as you get older, you have to learn to understand others. The things that used to frustrate you—you have to try to make sense of them, to put yourself in the other person’s place.”*

Another antagonistic figure then emerges—the “useless old man”—the opposite of the “active person” they strive to be. This idealized figure of the active person is not only wise and experienced but remains in motion, remains useful, and ultimately, remains alive: *“That’s the ‘useless old man,’ in quotes. That’s the ‘useless old man.’ But someone who stays active, a man who keeps busy, who comes home and says, ‘Look, I painted this, I hammered in this nail, I hung this picture’—you know he’s alive. But if he’s just sitting in a chair, watching the flies go by… I want to be a useful old man.”*

Overall, in this interview humor is used in several ways and in different rhetorical and ideological conversational contexts, but as in other interviews laughter is associated with accepting a viewpoint that the participants try to contradict, mitigate, or balance with their narrative asbout positive aging and the transmission of experience:

- *(Interviewer) What does aging mean to you—each of you? [10 s of silence].*
- *Just getting closer to death, that’s all. [Laughs]*

### 4.5 Comparative analysis

#### 4.5.1 Interplay of positioning and otherness

Across the four interviews, older adults position themselves in relation to others—family, younger generations, and institutions—while experiencing varying degrees of otherness. In the first and third groups, participants construct their identities primarily within the family, viewing younger generations as both a source of support and an unfamiliar other. Maintaining an “optimal distance” from relatives is key to balancing autonomy with emotional connection. In contrast, the second and fourth groups highlight societal exclusion and the institutional imposition of old age. The second group critiques how retirement and legal classifications render them invisible, while the fourth group emphasizes cultural attitudes that diminish their relevance, particularly for men. Masculinity emerges as a defining factor, with older men positioning themselves against the stereotype of the “useless old man,” instead aspiring to remain active and engaged. Despite these differences, a shared frustration runs across all groups: the absence of institutional spaces for intergenerational knowledge transfer reinforces their sense of marginalization. While some participants admire or envy youth, others critique their perceived lack of wisdom, attributing maturity solely to lived experience.

This sense of otherness is further shaped by normative discourse, which regulates expectations about aging and influences how older adults see themselves. In the first and third groups, self-imposed mandates emphasize positive aging—staying active, maintaining a good attitude, and accepting change. These take the form of personal mottos (“*One must always be happy”*) that reinforce a discourse of self-management. By contrast, the second and fourth groups focus on resisting externally imposed definitions of aging. The second group rejects legal and institutional categorizations (“*The law tells you at 60, and they push you off a cliff”*) as arbitrary constraints that strip them of their social roles. Meanwhile, the fourth group directs its normative discourse outward, demanding recognition as active contributors (*“We should have places to share our experiences with younger generations”*). Despite these distinct approaches, normativity in all groups functions as both self-regulation and negotiation—whether through internalized ideals, resistance to imposed identities, or advocacy for institutional support.

Laughter and humor emerge as key elements in how older adults navigate these tensions, acting as both coping mechanisms and discursive tools. In the first and third groups, humor strengthens social bonds, allowing participants—especially women—to reframe aging-related difficulties in a more manageable way. Laughter fosters solidarity and complicity, easing the weight of personal and collective challenges. However, in the second and fourth groups, humor functions differently. In the second group, laughter often marks abrupt thematic shifts, particularly when discussing death, signaling an attempt to evade discomfort. In the fourth group, humor serves as a means of asserting dominance in conversation, frequently surfacing as satirical remarks or critiques—at times taking a machista tone toward women’s appearances. While humor in this group fosters camaraderie, it also generates moments of tension, particularly for the interviewer. Across all groups, humor oscillates between reinforcing group identity, relieving tension, deflecting discomfort, and reinforcing social hierarchies.

#### 4.5.2 Interplay of humor and normativity

Older adults negotiate aging. In the first and third groups, where internalized norms emphasize positive aging, humor helps alleviate the pressure of these expectations. Laughter softens rigid mandates (“*One must always be happy”*), making them more socially acceptable while simultaneously reinforcing the obligation to remain active and autonomous. In contrast, in the second and fourth groups, humor becomes a tool for resisting external norms. In the second group, laughter undermines the legitimacy of institutional constraints, allowing participants to momentarily detach from their imposed roles. In the fourth group, humor reinforces masculinity by positioning the “active man” as an ideal in opposition to the “useless old man,” further entrenching gendered expectations around productivity and aging.

In all the interviews, humor plays multiple roles, appearing in different rhetorical and ideological contexts while shaping distinct narratives. Laughter frequently arises when participants recognize a viewpoint they attempt to contradict, soften, or reconcile—when they recognize a slight acceptance of the discourse of the other, marking these moments with particular intensity. Despite variations, a crucial role of humor every time the discourse of older adults yields in its resistance to discussing aging and death:

- *(Interviewer) What does aging mean to each of you? [10 s of silence].*
- *Just getting closer to death, that’s all. [Laughs]*

The dimensions of humor/laughter and normativity are closely interconnected, revealing both coping strategies and mechanisms of social regulation in the experience of aging. Three key relationships emerge. When normative discourse emphasizes self-imposed rules on how to age correctly, humor serves as a way to alleviate the pressure of these expectations. However, at the same time, using humor to reinforce these ideals can contribute to their naturalization, further solidifying the expectation to age in a “positive” and autonomous manner.

In the second and fourth groups, where normative discourse focuses on criticizing external impositions (from institutions, the law, or broader societal expectations), laughter operates as a form of distancing or resistance. In the second group, for instance, humor accompanies abrupt thematic shifts, signaling an attempt to evade imposed norms, such as mandatory retirement.

In some cases, laughter serves as a way to cope with a lack of recognition—such as when they lament being pushed aside by younger generations—while in others, it reinforces their role as active individuals (*“If you are just sitting in a chair, watching the flies go by…”*). In this sense, the interplay between normativity and humor delineates the boundaries between what is acceptable and what is questioned in the experience of aging.

In conclusion, humor and normativity intertwine in a dynamic of regulation and resistance. Laughter can both ease and reinforce aging norms, depending on whether it is used to accept, challenge, or negotiate the social structures that interviewees navigate. Humor and normativity intersect in shaping how older adults position themselves within their social world, and within the interview. Whether through humor as a means of negotiating intergenerational disconnection or normative discourse as a tool for structuring agency and resistance, both dimensions reveal the complexities of aging as a lived and discursive experience. The tension between self-imposed expectations and external categorizations, as well as between humor as a coping strategy and as a reinforcement of social norms, underscores the ongoing negotiation of identity, agency, and social belonging in later life.

## 5 Discussion

### 5.1 Discourse and laughter in aging

The findings presented here stem from an integrative analysis that explored multiple layers of discourse within each interview. Each conversation was approached as a case study, examining how laughter organized content and coordinated interaction within the specific interactional history of the dialogue. Our analysis followed a developmental logic, tracing how discursive positions and narrative differences emerged from layered interactional dynamics, whithout attempting a chronological reconstruction. This approach revealed two key patterns. First, partially shared discursive elements were mobilized differently across groups. Second, laughter functioned as a transversal resource for negotiating the unsaid—especially around mortality and dependency.

Rather than being merely an expressive or relational tool, humor served as a strategic discursive resource that opened temporary spaces for addressing what could not be directly confronted. Laughter functioned as a hinge between affect and normativity, enabling the articulation of tensions that otherwise remained implicit. Drawing on Bergson (2005), we argue that laughter in discourse may be treated as a symptom—a moment that both conceals and signals latent ideological conflict around what remains unsaid. This perspective aligns with Billig’s (1999) critical-discursive reading of laughter and the unconscious and resonates with Freud’s (1960) account of the comic as a site where psychic economy, social norms, and discursive form converge.

Humorous laughter in our data functioned symptomatically in multiple ways. It acted as a paralinguistic marker of censorship or affective ambiguity; a signal of evaluative intensity—ranging from joyful to degrading; a collective tool for managing discursive instability, whether through resistance or reinforcement; and a biosemiotic trace of the struggle between discursive order and affective overflow. In this sense, laughter events can be read as micro-revolts within the flow of discourse—bodily irruptions that unsettle the apparent stability of meaning, momentarily reconfiguring the relation between norm, body, and voice. As Bergson (2005) noted, laughter “indicates a slight revolt on the surface of social life” (p. 200), implying that these surface expressions resonate with deeper social movements. This view aligns with Voloshinov’s (1976) reading of Freud, which posits that what psychoanalysis treats as the unconscious is fundamentally ideological—constituted through social relations rather than individual psychic processes. Bergson’s work provides conceptual grounding for such an expansive, non-reductionist view of laughter as a bodily-discursive force.

Conducting ideological analysis through the lens of laughter provides a way to approach discourse beyond dualisms—particularly the divisions between meaning and affect, mind and body, or structure and expression. Our work proposes a path for a materialist critical discourse analysis, one that connects the historical sedimentation of discursive practices with the bodily intensity of utterances. In doing so, we bring into dialogue two theoretical traditions often treated as incompatible: the Marxian, Voloshinov-inspired conception of ideology as embedded in discourse, and a posthuman, neo-materialist view of affect as a force entangled with material-discursive practices. In our analysis, laughter stands at the intersection of these perspectives, making visible the affective labor of ideology and the ideological implications of affect.

This study proposes a discourse-analytical approach to laughter as a site where affect and ideology converge. Drawing from dialogical theory, we conceptualized discourse not as the transmission of content but as a field of positioning within socially and historically saturated scenes of interlocution. Within this framework, laughter was examined as a discursive symptom—an embodied enthymeme that condenses tensions, exposes implicit norms, and negotiates the boundaries of what can be said. Through the analysis of group interviews with older adults, we observed how laughter functioned not merely as a coping mechanism but as a subtle tool for managing the ideological tension between autonomy and dependency in aging discourse. This tension reflects broader debates about the relationship between frailty and successful aging (Pickard, 2023). When participants laughed at the prospect of physical decline or at being labeled “old,” they revealed unspoken cultural logics without articulating them directly, but by marking moments where personal experience confronted societal expectations. In this way, laughter provided a privileged entry point into the ideological dimensions of aging discourse. By bringing together Voloshinov’s concept of the social enthymeme with attention to embodied affect, we have demonstrated how social values around aging are not only spoken, but felt, negotiated, and transformed through seemingly peripheral communicative acts.

### 5.2 Aging, discourse, and the politics of subjectivity

Subjective aspects of aging are often framed in terms of challenges to well-being and cognitive ability, embedded within broader sociocultural dilemmas. From the active aging paradigm (Boudiny, 2013), agency and participation are seen as crucial dimensions of subjectivity in later life (Foster and Walker, 2015). Personal autonomy—such as performing daily tasks independently—is associated with fewer depressive symptoms (Bojorquez-Chapela et al., 2012), while social support and self-regulation strategies aid adaptation and psychological well-being (Hsu and Tung, 2010). While cognitive demands increase with age (Ennis et al., 2013), self-efficacy supports persistence in such tasks (Esposito et al., 2014). However, these analyses tend to overlook the lived subjective experience of aging, failing to account for the cultural and discursive construction of subjectivity.

Just as population aging is more than a demographic trend, individual aging is more than a psychobiological process. Each society defines who counts as old and prescribes what roles older individuals should or should not fulfill (Ekerdt et al., 2023; Majón-Valpuesta et al., 2016; McMunn et al., 2009), with recent Chilean research specifically highlighting how older adults navigate tensions between negative stereotypes and more positive, resistant narratives of aging (Pavez et al., 2023). In this sense, both aging narratives and the ways older adults construct themselves discursively are part of the broader social process by which bodily changes are rendered meaningful through discursive practices.

These discursive processes involve the configuration of evaluative positions toward aging. When participants laugh at the gap between their felt experience and societal expectations, or use humor to soften talk about mortality, they enact what Voloshinov (1976) called a “social enthymeme.” These moments of laughter act as embodied markers of ideological tension, revealing how older adults oscillate between resistance and acceptance of dominant aging narratives. Attending to such seemingly peripheral moments is key to understanding how age is constructed through discourse (Norrick, 2009).

Research on aging from a cultural standpoint has expanded into areas such as identity in modern societies (Norrick, 2009), spirituality and successful aging (Boswell and Boswell-Ford, 2010; Helmeke, 2006; Lewis, 2001; Lowis et al., 2011; Snodgrass and Sorajjakool, 2011), and intercultural variations in coping based on religion, worldview, or wisdom (Bailly and Roussiau, 2010; de Jager Meezenbroek et al., 2012; Hallaj et al., 2014; Perkins, 2010).

The question of subjectivity does not aim to uncover inner intimacy or collect evaluations; it interprets how older adults are affectively positioned through discursive operations. Our analysis of laughter as an ideological symptom exemplifies this, showing how older adults negotiate autonomy and dependence through subtle, embodied responses. When participants laugh at being called “granny” or at their own limitations, they engage in a performative negotiation of stereotypes. In contrast to views that reduce subjectivity to individual variation, we understand it as the capacity to speak and position oneself in discourse. This dialogical understanding aligns with our view of laughter as an embodied enthymeme—both making visible the tension between the said and the assumed in discourse. In addition, the dialogical approach enabled us to interpret participants’ positioning in the context of an interview conversation whose unfolding was unique and mediated by the interaction with the interviewer. Her reflective protocol was used as data to understand the affective context of discourse.

The comparative analysis of our four interview groups reveals patterns that echo recent findings on how older adults navigate aging discourses through humor and positioning strategies. Across groups, laughter often emerged at precisely those moments where participants encountered what Gilleard and Higgs (2020) call the “boundary” between the third and fourth age—points where autonomy confronts dependency, or where active aging ideals meet corporeal limitations. This boundary-marking function of laughter reflects broader patterns observed in studies of online humor among seniors (Nimrod and Berdychevsky, 2018), where jokes serve to negotiate social identities in relation to ageist stereotypes. Notably, our finding that laughter often accompanies moments of “accepting the discourse of the other” aligns with observations from studies of aging discourse and identity (Coupland, 2009; Ylänne, 2012) that older adults employ specific strategies when navigating stereotypes—including acknowledging but distancing themselves from negative age-related assumptions. The socioeconomic and gender differences we observed in humor patterns further support contemporary research on the intersectional nature of aging experiences (Grenier and Phillipson, 2018), highlighting how the ideological function of laughter varies according to one’s social positioning. For instance, upper-middle-class women’s laughter frequently reinforced “successful aging” norms of continued activity and autonomy, while middle-class men’s humor more often served to mask vulnerability—reflecting how diverse groups navigate what Lamb (2019) identifies as the class-inflected “moral project” of aging well.

How subjectivity is discursively articulated matters because discourse shapes how older adults’ lives and experiences materialize. Research in identity, aging, and regulation shows this clearly. For instance, Hilgeman et al. (2017) found that identity processes—accommodation, balance, and assimilation—predict beliefs about memory and self-efficacy more strongly than depression or perceived health. West and Hastings (2011) found that stronger beliefs in memory self-efficacy were linked to better cognitive performance. Jetten et al. (2009) showed that group recall of youth memories led to greater gains in memory tests than individual recall. These findings demonstrate that discourse influences functioning by constituting aging as a lived and embodied process.

We invite reflection on care practices, especially self-care, through the lens of discursive positioning. Care must attend to unspoken tensions shaping older adults’ discourse. When participants laugh at stereotypes like the “useless old man” or use humor to deflect dependency, they expose the ideological terrain of aging—a terrain often ignored by policy and care institutions. These findings align with recent research on precarious aging, which highlights how contemporary later life is increasingly defined by insecurity due to neoliberal policies, even as ‘successful aging’ rhetoric remains dominant (Grenier and Phillipson, 2018). Our analysis demonstrates that older adults’ seemingly peripheral communicative acts—especially laughter—constitute sophisticated performances of position-taking. Through laughter, they engage in nuanced ideological work, simultaneously reinforcing and resisting dominant narratives. The enthymematic quality of laughter offers a critical entry point for rethinking elder care in ways that recognize aging as dialogically constructed, affectively lived, and discursively negotiated.

## Data Availability

The raw data supporting the conclusions of this article will be made available by the authors, without undue reservation.
